# What Is the Environmental Impact of Wine Entering Global Value Chains? Studying the Evolution of CO_2_ Emissions from the Export of Spanish Denomination of Origin Wines

**DOI:** 10.3390/foods10071664

**Published:** 2021-07-19

**Authors:** Juan Sebastián Castillo-Valero, Inmaculada Carrasco, Marcos Carchano, Carmen Córcoles

**Affiliations:** 1 Institute of Regional Development, University of Castilla-La Mancha, 02071 Albacete, Spain; Sebastian.Castillo@uclm.es (J.S.C.-V.); Inmaculada.Carrasco@uclm.es (I.C.); 2 Faculty of Economics, University of Castilla-La Mancha, 02071 Albacete, Spain; carmen.corcoles@uclm.es

**Keywords:** CO_2_ emissions, carbon footprint, wine export, Spanish Denominations of Origin, multiregional input–output model

## Abstract

The continuous growth of the international wine trade and the expansion of international markets is having significant commercial, but also environmental, impacts. The benefits of vineyards in terms of ecosystem service provision are offset by the increase in CO_2_ emissions generated by transportation. Denominations of Origin, as quality labels, emphasise a wine’s links to the *terroir*, where specific elements of culture and environment merge together. However, Denominations of Origin can also have differentiating elements as regards environmental performance. Drawing on an extended multiregional input–output model applied to the Spanish Denominations of Origin with the largest presence in the international wine trade, this study shows that wines with the greatest exporting tradition are those that most reduced their carbon footprint per litre of exported wine in the period 2005–2018, thus being the most environmentally efficient.

## 1. Introduction

Wooded landscapes and perennial production systems provide benefits in terms of carbon sequestration and protecting biodiversity [1,2,3,4]. Thus, vineyards, as permanent woody crops, can provide ecosystem services (ES), such as maintaining soil fertility, retaining water and preserving landscapes [5] and have the capacity to generate ecosystems that allow for carbon storage, helping balance the carbon budget [6,7,8]. These functions are especially important in areas characterised by high levels of human pressure, such as suburban areas [5] and coastal areas [9]. For this reason, wine has long been considered an environmentally sound product [10,11,12], and little attention has been paid to its environmental impacts [12,13].

Much of wine production is organised under Denominations of Origin (DO), which allow unique ecosystems to be preserved and promote the diversity of species in their regions. They are quality labels granted to goods and services that include characteristics linked to their geographical origin. They are inspired by the ancient Mediterranean tradition (Greeks and Romans) of identifying products by their place of provenance when they share certain attributes.

Preserving the characteristics of *terroirs* has been one of the wine industry’s main concerns in defining production regions under the protected denomination of origin (DO) scheme. However, the wine market is now global. The evolution and changes in the consumption of agri-food products explain the variations in the supply chain and international trade [14] and their consequent environmental impacts. In recent decades, international trade has increased considerably, with an anthropogenic impact that has not been addressed in the majority of studies.

Agriculture is considered to be one of the most polluting activities on our planet. Changed land use and the implementation of large amounts of technical inputs (fuels, fertilisers, herbicides etc.) cause 24% of greenhouse gas emissions (GHG), of which 50% is accounted for by carbon dioxide (CO_2_) [6,15]. The expansion and intensification of agriculture is thus a threat to climate change [16] and biodiversity conservation [17,18,19,20,21] and produces changes in the earth’s systems [22,23].

More specifically, the presence of vineyards has expanded significantly in regions of the world with a Mediterranean climate, while many of the existing vineyards have intensified their production processes [24], with a consequent environmental impact. These trends increase with population growth and diet shifts [25] and decrease according to income [26]; a balance has not been achieved, despite improved productivity [27].

The beginning of the 21st century has seen a new geography of world trade emerge. It is characterised by the increased prominence of the southern regions, which has resulted in the integration of countries in Latin America and the Caribbean, Africa, Asia (including China) in trade flows that were dominated by the developed nations, many of them from the north. This has occurred thanks to processes of development and structural change in such countries, which permitted productive specialization, increased income and a rapid rise in consumption [28]. 

Thus, as well as the environmental impacts caused by the use and exploitation of land and the use of chemical products, fuels and water, we also have those produced by the increase in international trade [29] to which more than 60% of global environmental effects are related [30] and which are becoming increasingly significant [31]. More specifically, 14% of global greenhouse gas emissions are the result of freight transportation [32], with this being expected to rise by more than 50% by 2035 [33]. This situation is aggravated in the world of wine, where emissions from the distribution process represent 50% of the total emissions in the wine production life cycle [34,35,36,37]. 

In this context, the wine industry worldwide faces various socio-environmental problems and concerns [13,38,39], related, on the one hand, to the environmental impact generated by its activity [11,12,34,40,41,42,43] and, on the other, to underlying problems arising from climate change and the availability of natural resources such as water and energy [44]. For this reason, sustainability issues have gained importance in the sector [45,46,47,48] and become one of the main elements in the wine supply chain [49]. In this respect, consideration of environmental issues is increasingly identified with the quality elements of wine [50].

Consumers are paying increasing attention to sustainable wine products [51,52,53], pressing companies to use sustainable external (natural resource use) and internal (recycling, herbicide reduction and renewable energy use) environmental practices [54,55]. In fact, many studies have shown that the use of sustainable practices in wine production constitutes an important element in the purchase decisions of consumers [56,57,58,59,60,61,62], who are willing to pay higher prices for more sustainable products [11,49,63]. In this sense, wineries have begun to develop and implement new practices and environmental technologies [64], moving towards more sustainable cultivation and production techniques [65], with an increasing number of enterprises integrating sustainability in their activities [66,67].

Thus, the adoption of sustainable practices is consolidated as an element of differentiation in wineries [68,69,70], with specific practices such as eco-labels [56] that afford competitive advantages [53,71] and generate added value [54,72]. As there is a direct relationship between the adoption of such practices and the economic performance of companies [68,73,74], various authors [75] have shown that there is a growing marketing and business model orientation towards sustainability. Linking brand strategy to sustainability is becoming more and more frequent [76]. For our case study, appellations of origin, as a shared brand and quality mark, should be a source of sustainable competitive advantage [77] if they demonstrate improvements in the environmental impact of their wines.

The literature includes many works examining carbon and water footprints in the wine industry as indicators to guide the improvement of the environmental performance of wine production [78,79,80,81,82,83]. The most used standard for assessing the environmental impacts of products and processes is *life cycle assessment* (LCA) [84]. However, *life cycle costing* (LCC) [85] or simplified LCA approaches are also frequent, especially in small and medium-sized enterprises [86]. Initially developed as a decision-making tool, they involve amounts of multidimensional data, sometimes private, that are complex to understand, manage [83,87,88] and apply at meso- and macro-levels. Indeed, most studies focus on the footprint of production, using B2B (Business to Business) approaches, and thus exclude the distribution and the bottling and consumption stages [89].

An alternative way to calculate the environmental effects of international trade is through Multi-Regional Input–Output models that allow for complex assessments (including direct and indirect effects) with publicly accessible data, therefore making them reproducible. They also enjoy very wide regional coverage and enable the study of carbon leakage or the way in which impacts are distributed in different parts of the planet [90,91]).

Considering all of the above, the aim of this article is to make an original contribution to the literature by focusing on the evolution of the carbon footprint produced by the international trade and transportation of Spanish denomination of origin wines. To this end, we implemented a multiregional input–output model for the period 2005–2018, as reported by authors such as Ayuda et al. [92], Duarte et al. [93] and Duarte et al. [94]. To the best of our knowledge, this is the first study to apply this methodology to calculate the evolution of CO_2_ emissions produced by exports from the main Spanish DO regions. The findings may be significant for the commercial exploitation of DOs that present higher environmental efficiency, with pollution growing at lower levels than international trade.

The article is structured as follows: the next section describes denominations of origin and their development in different countries in greater detail. The third section explains the methodology used to calculate the CO_2_ emissions generated by Spanish wine exports, and the fourth section is devoted to presenting and discussing the results. The final section presents our conclusions.

## 2. Denominations of Origin as a Differentiation System

Denominations of origin represent integrated microsystems associated with quality schemes, which, in turn, are linked to regional and environmental settings, manifestations of culture and customs and productive specialisation. They encompass the singular characteristics of the physical environment and the land that underlies their agricultural production, where climate, geology, soil, topography and biology merge together. They are distinguished by their vegetation and their human capital, which help form the peculiarities of their wine production. They are also defined by different historic, economic and social circumstances, which have formed the respective winemaking sectors in each case. This combination of factors finally gives rise to the transferability of concepts such as quality and exclusivity transmitted in the end product [95,96,97].

The Spanish wine industry boasts 97 DOs (including Denominations of Origin, Qualified Denominations of Origin, Protected Geographical Indication wines and Single Estate wines), although 109 are contained in the European e-Bacchus database, which includes different nomenclature from the same regions (e.g., for reasons of language). This number is relatively small compared with those of the other two leading wine-producing countries, France and Italy, which are home to 432 and 474 PDOs, respectively. Despite this difference, wine DOs account for almost half the Denominations of Origin in Spain for the total number of agri-food products (a little more than 200).

Although since 2009 (under the most recent common organisation of the market in wine, Council Regulation (CE) n° 479/2008 of 29 April) the recognition of DOs has been unified in the sphere of the European Union, such figures were already regulated and recognised at a national level (dating back in Spain to the Spanish Wine Statute of 1933, under which the Denomination of Origin of Jerez was recognised in 1935; although Rioja had regulated its status in 1928, it was not consolidated unto 1947). However, precedents of protection of origin for wine go back to the 18th century in parts of Tuscany, including the recognition of Chianti, and for the Port Wine in Portugal, in 1756. Nonetheless, the impetus of quality label regulation is found in the 1883 signing of the Paris Convention for the Protection of Industrial Property, which was consolidated with the Madrid Agreement in 1891 (on the International Registration of Marks) and the 1958 Lisbon Agreement (for the Protection of Appellations of Origin and their International Registration), triggering the drive for protection of origin in the European countries that would subsequently form the Common Market and then the 12-member European Economic Community.

The key aspect for the definition of denominations of origin, which evidently affects their dimension and, therefore, their number, is the concept of *terroir*, which is the core element of the Denominations of Origin, linking it to a specific region, marked by natural factors (climate, latitude, altitude, daylight hours) and human factors, and the particularities and qualities of the wines they give rise to, as well as the economic impact of their recognition [98].

Although a long-established term associated with French traditions, the international definition of *terroir* was established by the International Organisation of Vine and Wine through resolution OIV/VITI/333/2010. The wine *terroir* “is a concept which refers to an area in which collective knowledge of the interactions between the identifiable physical and biological environment and applied viticultural and oenological practices develops, providing distinctive characteristics for the products originating from this area. Terroir includes specific soil, topography, climate, landscape characteristics and biodiversity features”.

In short, *terroir* encompasses specific characteristics of an area’s soil, topography, climate, landscape and biodiversity. It also adds a differential effect to the wine produced in that region, serving both domestic consumption and, especially, international trade [99].

Under DOs, actors from agricultural niche areas mobilise specific resources (biophysical environment, know-how, biodiversity, etc.) to emphasise the origin of food products [100]. Created in France and Italy at the beginning of the 20th century, they have now extended across the world [101] and underline the shift from production systems grounded in productivity to others based on quality [102].

Production under a quality label has social, legal and economic implications [103,104] and denotes elements of culture, identity and the environment [105,106]. Grape variety, [107] viticultural and oenological practices [108], climate conditions [109], geographical location of the vines [110], among other factors, shape the distinctive characteristics of an agri-food product such as wine. 

In the particular case of Spanish DOs, they only use the general, overarching name of the territory, space or area, and thus the number is smaller than in France and Italy. In France, denominations of origin take into account the existence of different wines, traditional terms that help underline their prestige, and grape varieties and also select more limited geographical locations as the expression of a *terroir* (municipalities or even small areas of land, *lieux-dits, climats*, etc.). Particularly significant is the exclusivity that distinguishes the most renowned and historically consolidated areas: Grands Crus/Premiers Crus from Burgundy (linked to specific plots of land) or the Grands Crus Classes from Bordeaux, identified with municipalities or châteaux [111]. In Italy, the pairing of geographical name and grape variety is widely used in the names of their Denominations of Origin [112].

It should be noted that the use of names in the concept of ‘denomination of origin’ in other wine-producing regions of the world (United States, Chile, South Africa, etc.) is far removed from the original European philosophy of differentiation by origin [113,114]. Moreover, the permanent Old–New World polemic between wine-producing countries with regard to trade-related aspects of intellectual property rights (TRIPS) and the debate on denominations of origin vs. collective names, is inclined towards the European exclusivity in terms and the longstanding respect for traditions in nomenclature in emerging countries [115].

Given the above context and the new challenge of climate change, “environmentally responsible wines” are set to be the new differentiating element to be included in denominations of origin, and thus it is essential to enhance the knowledge of the segment of transportation and distribution within the international wine trade [116].

## 3. Materials and Methods

The methodology used for this study is the extended multiregional input–output model (MIOM). Numerous works have used extended MIOMs for environmental analyses, among which it is worth noting those on the study of anthropogenic footprints of cities and regions [30,117], water footprints [92,93,94], the monetization of environmental impacts as a way to assess the effect of legislation on consumer mitigation [118] or studies that integrate data on companies with input–output data [119].

In a multiregional model, regions and/or countries are included with their own production technology, and trade is divided into intermediate and final, specifying the origin and destination (sector and country) of each good or productive sector considered. 

If we start from the Leontief elementary input–output quantity model, the basic equation can be expressed as follows:

(1) x=Ax + y

where x is the total output of the economy, y is the final demand, and A is the technical coefficient matrix. 

In a multiregional context, we can develop Equation (1) to consider the output of different regions as follows:

(2) $$\left(\begin{array}{c} \begin{array}{c} x^{1}\\ x^{2}\\ \ldots \\ x^{m} \end{array} \end{array}\right)=\left(\begin{array}{cccc} A^{11} & A^{12} & \ldots & A^{1m}\\ A^{21} & A^{22} & \ldots & A^{2m}\\ \ldots & \ldots & \ldots & \ldots \\ A^{m1} & A^{m2} & \ldots & A^{mm} \end{array}\right)\;*\;\left(\begin{array}{c} x^{1}\\ x^{2}\\ \ldots \\ x^{m} \end{array}\right)+\left(\begin{array}{c} y^{1}\\ y^{2}\\ \ldots \\ y^{m} \end{array}\right)$$

with x^r^ or y^r^ being vectors of output and final demand of each of the regions, and A^rr^, A^sr^ being the matrices of domestic and imported technical coefficients of each region. 

Equation (2) (like Equation (1)) can be solved using the Leontief inverse matrix, (I-A)^−1^, which, in the MIOM model will give us the direct and indirect requirements, both domestic and imported, demanded in the production of a specific sector of any country or region, per unit of final demand.

The input–output framework allows us to analyse different socioeconomic and environmental aspects by incorporating the factor we seek to study. Specifically, in this work, the factor required refers to the emissions data (E) for the sectors and countries included in the satellite accounts (information found in the MIOM database used in this work, the WIOD), which should be expressed in the model in terms of coefficients, that is, emissions per unit of production. As the aim of this work is to determine the emissions generated by Spanish exports of the different DO wines under study, the final demand vector will include these exports. The corresponding equation to be applied is the following:

(3) $$F=\;ê\;{\left(I-A\right)}^{\left(-1\right)}ŷ\;=Pŷ$$

where e is the emissions coefficient vector (E/x) and is diagonalised, in the same way as vector y, to obtain the results as a matrix, which yields richer results and allows for a deeper analysis.

In the case of two countries and two sectors, the matrix structure for the emissions multiplier is as follows:

(4) $$\left(\begin{array}{ccccc} {ɛ}_{11}^{11} & {ɛ}_{12}^{11} & \ldots \; & {ɛ}_{21}^{12} & {ɛ}_{12}^{12}\\ {ɛ}_{21}^{11} & {ɛ}_{22}^{11} & \ldots \; & {ɛ}_{21}^{12} & {ɛ}_{22}^{12}\\ \ldots & \ldots & \ldots \; & \ldots & \ldots \\ {ɛ}_{11}^{21} & {ɛ}_{12}^{21} & \ldots \; & {ɛ}_{11}^{22} & {ɛ}_{11}^{22}\\ {ɛ}_{21}^{21} & {ɛ}_{22}^{21} & \ldots \; & {ɛ}_{21}^{22} & {ɛ}_{22}^{22} \end{array}\right)=\;\left(\begin{array}{cc} p^{11} & p^{12}\\ p^{21} & p^{22} \end{array}\right)$$

where ɛ^rs^_ij_ shows the emissions of sector “i” from country “r” to satisfy a unit of final demand of sector “j” from country “s”.

Thus, observing matrix *p* by rows, we have the emissions resulting from the production process of a good, while the columns show the emissions incorporated into the production process through the inputs used in manufacturing the product.

In this case, we multiply *p* by the vector of the Spanish wine exports to each of the countries, for each DO under study (being vector y), following the example above (two countries, two sectors) and, by columns, we obtain the emissions generated by these exports in each country and sec La nota a pie de página no está permitida en este diario, por lo que la hemos trasladado al texto, por favor confirme el texto completo.tor.

(5) $$\left(\begin{array}{cc} p^{11} & p^{12}\\ p^{21} & p^{22} \end{array}\right)*\left(\begin{array}{cc} y^{11} & 0\\ 0 & p^{22} \end{array}\right)=\left(\begin{array}{ccccc} p^{11} & y^{11} & \ldots \; & p^{12} & p^{22}\\ \ldots \; & \ldots \; & \ldots \; & \ldots \; & \ldots \;\\ p^{21} & y^{11} & \ldots \; & p^{22} & p^{22} \end{array}\right)$$

To calculate the environmental impact of Spanish DO wine exports for the period 2005–2016, we used three datasets: data provided by the most recent version of the World Input–Output Database (WIOD) [120], from which we obtained the input–output tables for 44 regions and 56 sectors, available up to 2014 (for the 2016 calculations, we used the WIOD data for 2014 (the most recent available) under the assumption that there were no structural changes in the sector); data taken from the same source on emissions expressed in kilotonnes of CO_2_; finally, data provided by the Spanish ministry of Agriculture, Fisheries and Food [121] on the value of wine exports in millions of euros at sale prices for the different DOs considered. The results of the current work are presented using the same units as in the databases (Mill. US$ and ktCO_2_). The technical coefficients are those of the manufacture of food products, beverages and tobacco products. The calculations were performed using the latest version of MATLAB.

## 4. Results and Discussion

**Figure 1:**
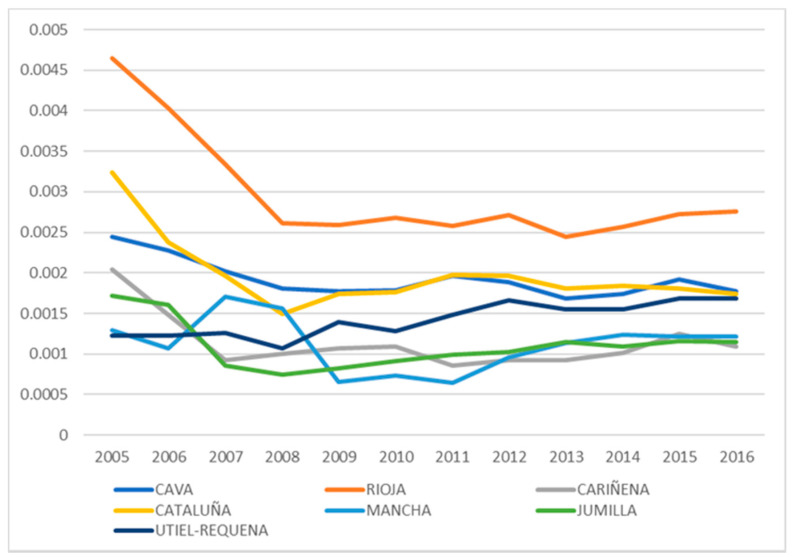
Evolution of CO_2_ emissions per exported litre of wine for the main Spanish DOs.

For an in-depth study of consumption-based emissions, we focused on the recent variations in the carbon footprint of DO wine exports in the period 2005–2016. The seven denominations included in the sample (Cava, Rioja, Cariñena, Cataluña, La Mancha, Jumilla and Utiel-Requena) account for 66% of exports (by volume), with Cava being the highest exporter at 24%, followed by Rioja (21%), Cariñena (6%), Cataluña (5%), Mancha (4%), Jumilla (3%) and Utiel-Requena (3%). The calculation of the CO_2_ emissions generated per litre of exported wine for each denomination of origin shows the differences between the seven DOs (Figure 1). With the exports growing in all cases (except La Mancha), the emissions generated per litre exhibit a different behaviour in each denomination, falling in the cases of Rioja, Cataluña, Cava, Cariñena and Jumilla, with the reduction being the most intense for the first two denominations, while emissions generated by La Mancha and Utiel-Requena increased between 2005 and 2016.

The evolution in the emission coefficients in different countries and transport sectors (land transport and water transport, which are two most widely used) (Appendix A) may have contributed to the fall in the emissions generated per litre of exported wine. In this sense, two aspects can be underlined: on the one hand, taking into account that most exports within the European Union are distributed by land, emissions fell thanks to efficiency gains in the use of this type of transport, with a decrease in the emissions coefficient in the land transport sector for all the countries considered; on the other hand, taking into account the movement of exports from the European Union to the rest of the world, where wine is mainly transported by ship, efficiency gains are also observed, with a decrease in the emissions coefficient of the sector (water transport) in all the countries, which contributes to the fall in emissions generated by the exports of DO wine.

**Figure 2:**
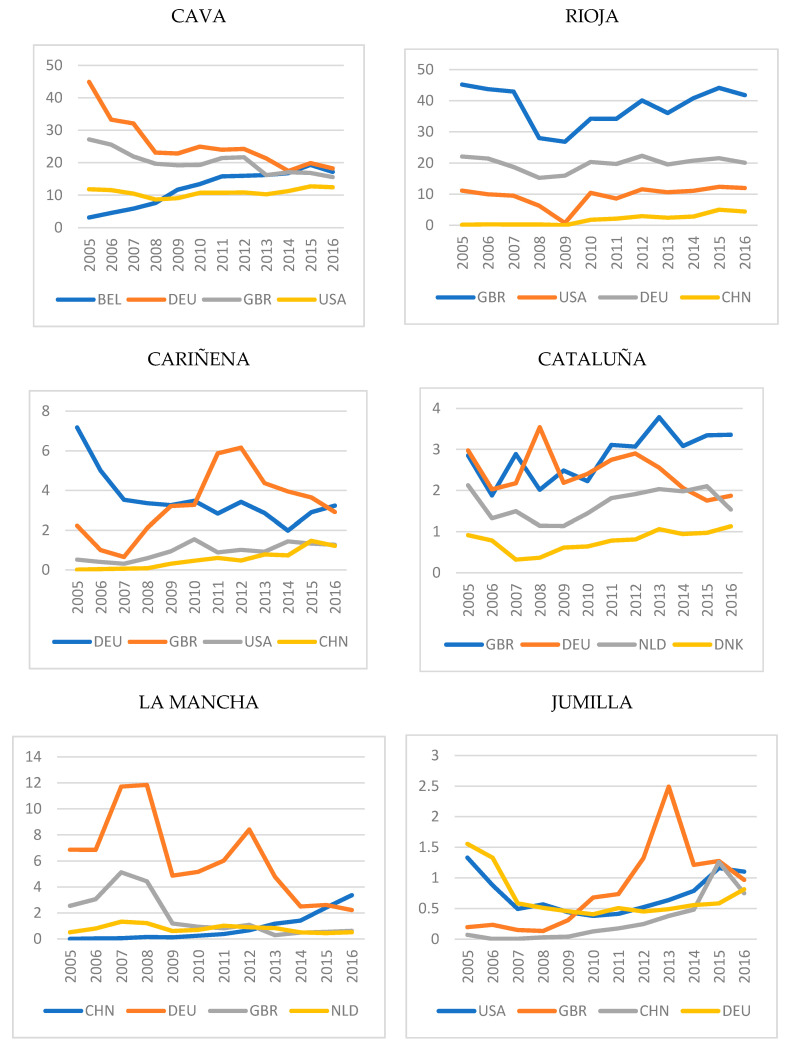

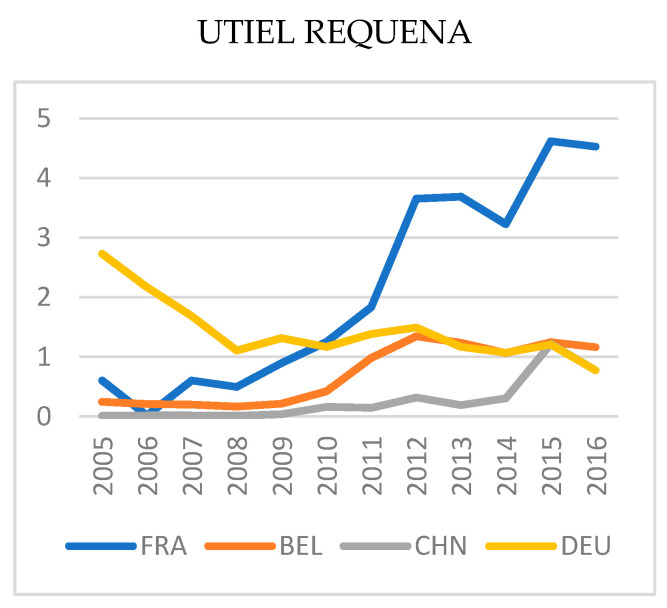
Evolution of CO_2_ emissions generated by Spanish wine exports from each DO in relation to the most significant importing countries.

As regards the study of the environmental impact by country, Figure 2 shows the evolution of the emissions generated in the export destinations with the largest carbon footprint derived from the wine exports from each of the denominations of origin. Again, different behaviours can be observed according to each DO. For example, in the case of Cava, it can be seen that emissions fell in the period 2005–2016 in the main importing countries, with the exception of Belgium, while in the case of Cataluña, emissions increased in the United Kingdom and Denmark and fell in Germany and the Netherlands. Thus, differences can also be observed in the by-country analysis of emissions generated by the exports of the different DOs. The only common pattern found in all cases is the decline in emissions in recent years, with the exception of particular cases, such as Denmark for the DO of Cataluña, China for the DO of La Mancha, and Germany for the DO of Jumilla.

In short, the countries that most contribute to the carbon footprint of the Spanish DO wine exports under study are Germany, United Kingdom, United States and China. It is worth underlining the substantial growth in wine imported from Spain, given that the country was a later entrant in the wine market. 

**Figure 3:**
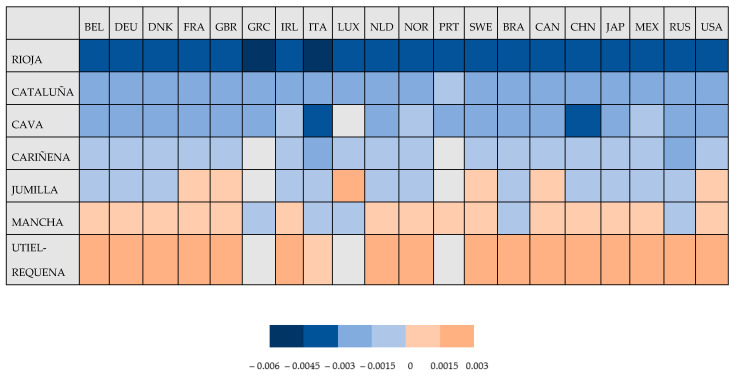
Variation in the CO_2_ emissions per litre of exported wine by DO in the destination countries (2005–2016).

Figure 3 shows the variation in 2005–2016 (in order to avoid disruptions, the first year is considered as the mean of 2005, 2006, 2007, while the last year is considered as the mean of 2014, 2015, 2016) in the ratio of emissions per litre of each DO in the different importing countries. Countries where emissions fell are in blue, countries where emissions increased are in orange, and those without a significant variation in the denomination of origin are in grey. The darker the colour, the more intense is the variation.

The denominations of origin with the largest fall in emissions per litre of exported wine were Rioja, Cataluña and Cava, with Rioja being the DO for which the decline was the strongest, due to the greater decrease in emissions generation per litre in Italy, Russia and China, the main countries where emissions grew proportionally less than exports (see Appendix B). Following Rioja are Cataluña and Cava, for which the fall is fundamentally explained by the lower contribution of emissions by Italy in both cases. The decline in emissions corresponding to Cariñena and Jumilla was less intense than in the previous cases. The last places are occupied by La Mancha and Utiel-Requena, denominations for which the CO_2_ emissions increased, and the variation in the ratio of emissions per litre is positive. For the former DO, the reason can be found in the behaviour of countries such as China, France, United Kingdom, Japan, Mexico, Netherlands and the United States, in all of which the emissions grew more than the exports. In the latter case, that of Utiel-Requena, the reason can be found in the behaviour of Belgium, Brazil, Canada, China, the Netherlands, Sweden and the United States, where, as in the case of the La Mancha DO, the emissions generated in these countries proportionally increased more than exports.

Our findings are consistent with those of Bolea et al. [29], Wiedmann and Lenzen [30], López et al. [31] and Avetisyan [32], as they confirm that the increase in international wine trade causes CO_2_ emissions to increase, which logically counteracts the beneficial effects reported in the literature of vineyards in terms of ecosystem services. Our study, however, allows us to qualify the previous findings as we show that the international trade of the various DOs has different environmental impacts. This differential integration of each DO in global value chains, and the consequent environmental impacts, is undeniably the result of changes in preferences and consumption that drive the evolution of international trade supply chains, as suggested by Weinzettel et al. [14]. Consequently, previous literature [45,46,47,48], has revealed that sustainability issues are becoming more and more important in the world of wine and one of the main elements in the wine supply chain [49]. 

Our findings also show the shift of exports towards the south, with an increased volume of exports to countries such as China, Brazil and Japan, thus confirming the notion that, in general terms, the new geography of global trade is characterised by the rise in prominence in trade flows of the regions of the south [28]. Additionally, the results confirm the expansion of trade towards more distant countries such as Russia, Canada, the United State and Mexico and the relative loss of the importance of exports to closer European countries (which continue to be, nonetheless, the main destinations of trade). However, the access to these more distant countries appears not to have environmental consequences, as the variation in CO_2_ emissions per litre is not homogenous, being more closely linked to the general behaviour of each DO. 

These results may be relevant for DOs that demonstrate the best environmental performance as integrated micro-systems associated with quality schemes and in line with literature showing that environmental aspects are increasingly identified with the quality elements of wine [50]. The results obtained in this study can be interpreted more broadly, allowing an important conclusion to be drawn in the case of DOs that demonstrate a reduction in emissions per litre, as this result can be used as a differential element in wine [68,69,70], which can be specified through instruments such as eco-labels [56], thus favouring the development of competitive advantages [53,71,77] and the generation of added value [54,72]. Concern for the environment shown by increasingly larger groups of consumers can undoubtedly serve as a source of competitive advantages for DOs that are able to exhibit greater environmental efficiency [77].

## 5. Conclusions

Given the opening of a debate in the European Union on the need for a carbon border adjustment mechanism in the international agri-food trade and the possibility of the beginnings of climate dumping by countries that are less proactive in the fight against climate change, it is essential to conduct research like the present one on wine trade, that can serve as a reference to establish a new order of international trade centred on the environment.

The prototypical European Union concept of wine differentiated by its territory of origin should advance hand in hand with methods that foster a new approach to the international wine trade, look to the environment (greater bulk production, fermentation and/or packaging/bottling at the destination, multilocation of industry and distribution, etc.) and should be able to take their part in the environmental commitment undertaken by the European Union in its Green Deal and help achieve carbon neutrality in 2050.

In this sense, our study reinforces the evidence that changes in export destinations, diversification and more remote destinations (compatible with most Spanish wine trade being concentrated in the European Union) are no barrier to significantly enhancing the environmental efficiency of the exports of certain denominations of origin.

We can highlight first the fall in CO_2_ emissions per litre generated by Spanish wine exports for the DO with the greatest proportion of foreign trade, namely, Cava, Rioja, Cariñena, Cataluña and Jumilla, with the drop being greatest for Rioja. Only for the DOs of La Mancha and Utiel-Requena, for which export volume and tradition are less significant, was there a rise in the carbon footprint per litre. Second, in the study on the countries where wine exports from each of the Spanish DOs generate a greater footprint, a general decrease in emissions over recent years was identified. The countries that generated most emissions related to all the Spanish DOs were Germany, Great Britain, the United States and China. 

In the period under analysis (2005–2016), the variation in the rate of emissions per litre in the different importing countries showed that, considering the denominations of origin for which the fall in emissions was greatest, Italy was the best-performing country (the emissions grew proportionally less than the exports). In the case of the DOs where emissions per litre rose (La Mancha and Utiel-Requena), China, the Netherlands and the Unites States are the countries that most contributed to this increase, as the emissions generated grew proportionally more than the exports.

This work has important consequences for academics and practitioners: first of all, for academics, as a first attempt to measure the carbon footprint associated with international freights of the main exporting Spanish DO and its evolution. The input–output method serves to complement others such as Life Cycle Assessment. Secondly, for practitioners, as we provide data and calculations demonstrating the earnings in CO_2_ emissions of Spanish DO that they can take into account in their firms’ business models in order to take competitive advantages by exhibiting their greater environmental efficiency by means of eco-labelling or certification schemes.

This analysis opens new avenues for further qualitative research on the most effective way to implement carbon border adjustment mechanisms. It also suggests the possibility of studying the viability of voluntary carbon credit markets (which include all stages of the food chain, that is, production, transformation and distribution) or the design of marketing strategies to attract new consumers, such as millennials and/or centennials, who are much more sensitive to environmentally responsible agri-food products. The denominations of origin have the opportunity to exploit their gains in environmental efficiency and to make them part of the intrinsic value associated with the quality label.

## Figures and Tables

**Figure A1:**
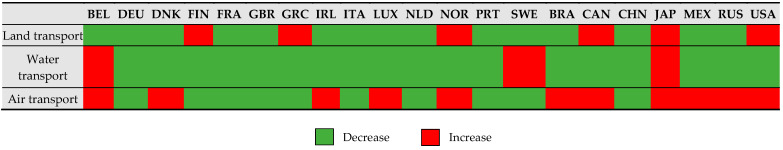
Difference in the CO_2_ emissions coefficient by transport sector (2005–2016). Source: own preparation based on Timmer, M.P., Dietzenbacher, E., Los, B, Stehren and de Vries, G.J. (2015).

## Appendix B

**Table A1:** Absolute variation in emissions per litre generated by Spanish DO wine exports (2005–2016). Source: Own preparation. * Non data available.

	BEL	DEU	DNK	FRA	GBR	GRC	IRL	ITA	LUX	NLD	NOR	PRT	SWE	BRA	CAN	CHN	JAP	MEX	RUS	USA
RIOJA	−3.4 × 10^−5^	−3.3 × 10^−5^	−3.4 × 10^−5^	−3.2 × 10^−5^	−3.3 × 10^−5^	−5.4 × 10^−5^	−3.4 × 10^−5^	−5.3 × 10^−5^	−4.1 × 10^−5^	−3.4 × 10^−5^	−3.3 × 10^−5^	−3.0 × 10^−5^	−3.3 × 10^−5^	−3.6 × 10^−5^	−3.2 × 10^−5^	−3.8 × 10^−5^	−3.4 × 10^−5^	−3.8 × 10^−5^	−3.9 × 10^−5^	−3.3 × 10^−5^
CATALUÑA	−1.7 × 10^−5^	−1.6 × 10^−5^	−1.7 × 10^−5^	−1.5 × 10^−5^	−1.6 × 10^−5^	−2.9 × 10^−5^	−1.7 × 10^−5^	−2.9 × 10^−5^	−2.2 × 10^−5^	−1.6 × 10^−5^	−1.6 × 10^−5^	−1.3 × 10^−5^	−1.6 × 10^−5^	−1.7 × 10^−5^	−1.6 × 10^−5^	−1.9 × 10^−5^	−1.6 × 10^−5^	−1.9 × 10^−5^	−2.0 × 10^−5^	−1.6 × 10^−5^
CAVA	−2.4 × 10^−5^	−2.5 × 10^−5^	−2.2 × 10^−5^	−2.2 × 10^−5^	−2.5 × 10^−5^	−1.9 × 10^−5^	−1.3 × 10^−5^	−3.8 × 10^−5^	NA *	−2.5 × 10^−5^	−1.4 × 10^−5^	−2.7 × 10^−5^	−2.5 × 10^−5^	−2.2 × 10^−5^	−2.1 × 10^−5^	−3.2 × 10^−5^	−2.3 × 10^−5^	−1.0 × 10^−5^	−2.7 × 10^−5^	−2.7 × 10^−5^
CARIÑENA	−1.2 × 10^−5^	−1.2 × 10^−5^	−1.2 × 10^−5^	−1.1 × 10^−5^	−1.2 × 10^−5^	NA *	−1.2 × 10^−5^	−2.0 × 10^−5^	−1.5 × 10^−5^	−1.2 × 10^−5^	−1.2 × 10^−5^	NA *	−1.1 × 10^−5^	−8.7 × 10^−6^	−1.1 × 10^−5^	−5.9 × 10^−6^	−1.2 × 10^−5^	−1.4 × 10^−5^	−1.7 × 10^−5^	−1.1 × 10^−5^
JUMILLA	−2.3 × 10^−7^	−6.5 × 10^−8^	−3.6 × 10^−7^	6.3 × 10^−7^	5.9 × 10^−8^	NA *	−6.3 × 10^−7^	−8.3 × 10^−6^	1.5 × 10^−5^	−7.3 × 10^−8^	−1.6 × 10^−7^	NA *	2.3 × 10^−7^	−5.3 × 10^−7^	3.2 × 10^−7^	−3.1 × 10^−6^	−1.2 × 10^−7^	−1.6 × 10^−6^	−2.0 × 10^−6^	1.6 × 10^−7^
MANCHA	1.6 × 10^−6^	1.8 × 10^−6^	1.8 × 10^−6^	2.3 × 10^−6^	1.9 × 10^−6^	−8.7 × 10^−6^	1.8× 10^−6^	−5.6 × 10^−6^	−3.1 × 10^−6^	1.6 × 10^−6^	1.7 × 10^−6^	3.7 × 10^−6^	2.2 × 10^−6^	−2.4 × 10^−7^	2.2 × 10^−6^	2.1 × 10^−6^	1.6 × 10^−6^	1.2 × 10^−7^	−3.4 × 10^−7^	2.0 × 10^−6^
UTIEL-REQUENA	1.8 × 10^−5^	1.8 × 10^−5^	1.8 × 10^−5^	1.9 × 10^−5^	1.9 × 10^−5^	NA*	1.8 × 10^−5^	8.1 × 10^−6^	NA*	1.8 × 10^−5^	1.8 × 10^−5^	NA *	1.9 × 10^−5^	1.6 × 10^−5^	1.9 × 10^−5^	1.6 × 10^−5^	1.8 × 10^−5^	1.6 × 10^−5^	1.6 × 10^−5^	1.9 × 10^−5^

## References

[1] Williams J.N., Hollander A.D., O’Geen A.T., Thrupp L.A., Hanifin R., Steenwerth K., McGourty G., Jackson L.E. (2011). Assessment of carbon in woody plants and soil across a vineyard-woodland landscape. Carbon Balance Manag..

[2] Albrecht A., Kandji S.T. (2003). Carbon sequestration in tropical agroforestry systems. Agric. Ecosyst. Environ..

[3] Hergoualc’H K., Verchot L. (2011). Stocks and fluxes of carbon associated with land use change in Southeast Asian tropical peatlands: A review. Glob. Biogeochem. Cycles.

[4] Murdiyarso D., van Noordwijk M., Wasrin U., Tomich T., Gillison A. (2002). Environmental benefits and sustainable land-use options in the Jambi transect, Sumatra. J. Veg. Sci..

[5] Brunori E., Farina R., Biasi R. (2016). Sustainable viticulture: The carbon-sink function of the vineyard agro-ecosystem. Agric. Ecosyst. Environ..

[6] Tezza L., Vendrame N., Pitacco A. (2019). Disentangling the carbon budget of a vineyard: The role of soil management. Agric. Ecosyst. Environ..

[7] Asbjornsen H., Hernandez-Santana V., Liebman M., Bayala J., Chen J., Helmers M., Ong C.K., Schulte L.A. (2014). Targeting perennial vegetation in agricultural landscapes for enhancing ecosystem services. Renew. Agric. Food Syst..

[8] Williams J.N., Morandé J.A., Vaghti M.G., Medellín-Azuara J., Viers J.H. (2020). Ecosystem services in vineyard landscapes: A focus on aboveground carbon storage and accumulation. Carbon Balance Manag..

[9] Biasi R., Barbera G., Marino E., Brunori E., Nieddu G. (2010). Viticulture as crucial cropping system for counteracting the desertification of coastal land. XXVIII International Horticultural Congress on Science and Horticulture for People (IHC2010).

[10] Russell A., Battaglene T. (2007). Trends in Environmental Assurance in Key Australian Wine Export Markets.

[11] Barber N., Taylor C., Strick S. (2009). Wine consumers’ environmental knowledge and attitudes: Influence on willingness to purchase. Int. J. Wine Res..

[12] Gabzdylova B., Raffensperger J.F., Castka P. (2009). Sustainability in the New Zealand wine industry: Drivers, stakeholders and practices. J. Clean. Prod..

[13] Ene S.A., Teodosiu C., Robu B., Volf I. (2013). Water footprint assessment in the winemaking industry: A case study for a Romanian medium size production plant. J. Clean. Prod..

[14] Weinzettel J., Vačkářů D., Medková H. (2019). Potential net primary production footprint of agriculture: A global trade analysis. J. Ind. Ecol..

[15] Smith P., Bustamante M., Ahammad H., Clark H., Dong H., Elsiddig E.A., Haberl H., Harper R., House J., Jafari M., Edenhofer O., Pichs-Madruga R., Sokona Y., Farahani E., Kadner S., Seyboth K., Adler A., Baum I., Brunner S., Eickemeier P. (2014). Agriculture, forestry and other land use (AFOLU). Climate Change 2014: Mitigation of Climate Change: Contribution of Working Group III to the Fifth Assessment Report of the Intergovernmental Panel on Climate Change.

[16] Foley J.A., Ramankutty N., Brauman K.A., Cassidy E.A., Gerber J.S., Johnston M., Mueller N.D., O’Connell C., Ray D.K., West P.C. (2011). Solutions for a cultivated planet. Nature.

[17] Tscharntke T., Clough Y., Wanger T.C., Jackson L., Motzke I., Perfecto I., Vandermeer J., Whitbread A. (2012). Global food security, biodiversity conservation and the future of agricultural intensification. Biol. Conserv..

[18] Paiola A., Assandri G., Brambilla M., Zottini M., Pedrini P., Nascimbene J. (2020). Exploring the potential of vineyards for biodiversity conservation and delivery of biodiversity-mediated ecosystem services: A global-scale systematic review. Sci. Total. Environ..

[19] Green R.E., Cornell S.J., Scharlemann J.P.W., Balmford A. (2005). Farming and the Fate of Wild Nature. Science.

[20] Katayama N., Bouam I., Koshida C., Baba Y.G. (2019). Biodiversity and yield under different land-use types in orchard/vineyard landscapes: A meta-analysis. Biol. Conserv..

[21] Winter S., Bauer T., Strauss P., Kratschmer S., Paredes D., Popescu D.M., Landa B., Guzmán G., Gómez J.A., Guernion M. (2018). Effects of vegetation management intensity on biodiversity and ecosystem services in vineyards: A meta-analysis. J. Appl. Ecol..

[22] Mace G.M., Reyers B., Alkemade R., Biggs R., Chapin F.S., Cornell S., Díaz S., Jennings S., Leadley P., Mumby P. (2014). Approaches to defining a planetary boundary for biodiversity. Glob. Environ. Chang..

[23] Rockström J., Steffen W., Noone K., Persson A., Chapin F.S., Lambin E.F., Lenton T.M., Scheffer M., Folke C., Schellnhuber H.J. (2009). A safe operating space for humanity. Nature.

[24] Caraveli H. (2000). A comparative analysis on intensification and extensification in mediterranean agriculture: Dilemmas for LFAs policy. J. Rural Stud..

[25] Alexander P., Rounsevell M., Dislich C., Dodson J.R., Engström K., Moran D. (2015). Drivers for global agricultural land use change: The nexus of diet, population, yield and bioenergy. Glob. Environ. Chang..

[26] Tilman D., Balzer C., Hill J., Befort B.L. (2011). Global food demand and the sustainable intensification of agriculture. Proc. Natl. Acad. Sci. USA.

[27] Ray D.K., Mueller N.D., West P., Foley J.A. (2013). Yield Trends Are Insufficient to Double Global Crop Production by 2050. PLoS ONE.

[28] Horner R., Nadvi K. (2017). Global value chains and the rise of the Global South: Unpacking twenty-first century polycentric trade. Glob. Netw..

[29] Bolea L., Duarte R., Sánchez-Chóliz J. (2020). Exploring carbon emissions and international inequality in a globalized world: A multiregional-multisectoral perspective. Resour. Conserv. Recycl..

[30] Wiedmann T., Lenzen M. (2018). Environmental and social footprints of international trade. Nat. Geosci..

[31] López L.A.L., Cadarso M.Á., Ortiz M. (2020). La huella de carbono del comercio internacional español. ICE, Revista de Economía.

[32] Avetisyan M. (2018). Impacts of global carbon pricing on international trade, modal choice and emissions from international transport. Energy Econ..

[33] IPCC Climate Change (2014). Mitigation of Climate Change. Contribution of Working Group III to the Fifth Assessment Report of the Intergovernmental Panel on Climate Change Cambridge.

[34] Cholette S., Venkat K. (2009). The energy and carbon intensity of wine distribution: A study of logistical options for delivering wine to consumers. J. Clean. Prod..

[35] Colman T., Päster P. (2009). Red, white, and ‘green’: The cost of greenhouse gas emissions in the global wine trade. J. Wine Res..

[36] Point E., Tyedmers P., Naugler C. (2012). Life cycle environmental impacts of wine production and consumption in Nova Scotia, Canada. J. Clean. Prod..

[37] Neto B., Dias A.C., Machado M. (2013). Life cycle assessment of the supply chain of a Portuguese wine: From viticulture to distribution. Int. J. Life Cycle Assess..

[38] Atkin T., Gilinsky A., Newton S.K. (2012). Environmental strategy: Does it lead to competitive advantage in US wine industry?. Int. J. Wine Bus. Res..

[39] Christ K.L., Burritt R.L. (2013). Critical environmental concerns in wine production: An integrative review. J. Clean. Prod..

[40] Maicas S., Mateo J.J. (2020). Sustainability of Wine Production. Sustainability.

[41] Marshall R.S., Cordano M., Silverman M. (2005). Exploring individual and institutional drivers of proactive environmentalism in the US Wine industry. Bus. Strat. Environ..

[42] Smyth M., Nesbitt A. (2014). Energy and English wine production: A review of energy use and benchmarking. Energy Sustain. Dev..

[43] Vázquez-Rowe I., Rugani B., Benetto E. (2013). Tapping carbon footprint variations in the European wine sector. J. Clean. Prod..

[44] Gilinsky A., Newton S.K., Vega R.F. (2016). Sustainability in the global wine industry: Concepts and cases. Agric. Agric. Sci. Procedia.

[45] Santiago-Brown I., Jerram C., Metcalfe A., Collins C. (2015). What Does Sustainability Mean? Knowledge Gleaned from Applying Mixed Methods Research to Wine Grape Growing. J. Mix. Methods Res..

[46] Chiusano L., Cerutti A., Cravero M.C., Bruun S., Gerbi V. (2015). An Industrial Ecology approach to solve wine surpluses problem: The case study of an Italian winery. J. Clean. Prod..

[47] Corbo C., Lamastra L., Capri E. (2014). From Environmental to Sustainability Programs: A Review of Sustainability Initiatives in the Italian Wine Sector. Sustainability.

[48] Remaud H., Muelles S., Chvyl P., Lockshin L. Do Australian wine consumers value organic wine?. Proceedings of the 4th International Conference of the Academy of Wine Business Research.

[49] Forbes S.L., Cohen D.A., Cullen R., Wratten S.D., Fountain J. (2009). Consumer attitudes regarding environmentally sustainable wine: An exploratory study of the New Zealand marketplace. J. Clean. Prod..

[50] Bruwer J., Alant K. (2009). The hedonic nature of wine tourism consumption: An experiential view. Int. J. Wine Bus. Res..

[51] Bonn M.A., Cronin J.J., Cho M. (2016). Do environmental sustainable practices of organic wine suppliers affect consumers’ behavioral intentions? The moderating role of trust. Cornell Hosp. Q..

[52] Pomarici E., Vecchio R. (2014). Millennial generation attitudes to sustainable wine: An exploratory study on Italian consumers. J. Clean. Prod..

[53] Dressler M., Paunović I. (2019). Towards a conceptual framework for sustainable business models in the food and beverage industry. Br. Food J..

[54] Pullman M.E., Maloni M.J., Dillard J. (2010). Sustainability Practices in Food Supply Chains: How is Wine Different?. J. Wine Res..

[55] Szolnoki G. (2013). A cross-cultural comparison of sustainability in the wine-industry. J. Clean. Prod..

[56] Berghoef N., Dodds R. (2011). Potential for sustainability eco-labeling in Ontario’s wine industry. Int. J. Wine Bus. Res..

[57] Zucca G., Smith D.E., Mitry D.J. (2009). Sustainable viticulture and winery practices in California: What is it, and do customers care. Int. J. Wine Res..

[58] Vecchio R. (2013). Determinants of willingness-to-pay for sustainable wine: Evidence from experimental auctions. Wine Econ. Policy.

[59] D’Souza C., Taghian M., Lamb P. (2006). An empirical study on the influence of environmental labels on consumers. Corp. Commun. Int. J..

[60] Schäufele I., Hamm U. (2017). Consumers’ perceptions, preferences and willingness-to-pay for wine with sustainability characteristics: A review. J. Clean. Prod..

[61] Szolnoki G., Hauck K. (2020). Analysis of German wine consumers’ preferences for organic and non-organic wines. BFJ.

[62] Pomarici E., Vecchio R., Mariani A. (2015). Wineries’ Perception of Sustainability Costs and Benefits: An Exploratory Study in California. Sustainability.

[63] Sogari G., Mora C., Menozzi D. (2016). Factors driving sustainable choice: The case of wine. Br. Food J..

[64] Galbreath J., Charles D., Oczkowski E. (2016). The Drivers of Climate Change Innovations: Evidence from the Australian Wine Industry. J. Bus. Ethic.

[65] Soosay C., Fearne A., Dent B. (2012). Sustainable value chain: A case study of Oxford Landing. Supply Chain Manag..

[66] Evans P.B. (2012). Embedded Autonomy: States and Industrial Transformation.

[67] Richter B., Hanf J. (2021). Cooperatives in the Wine Industry: Sustainable Management Practices and Digitalisation. Sustainability.

[68] Annunziata E., Pucci T., Frey M., Zanni L. (2018). The role of organizational capabilities in attaining corporate sustainability practices and economic performance: Evidence from Italian wine industry. J. Clean. Prod..

[69] Grimstad S. (2011). Developing a framework for examining business-driven sustainability initiatives with relevance to wine tourism clusters. Int. J. Wine Bus. Res..

[70] Signori P., Flint D.J., Golicic S.L. (2017). Constrained innovation on sustainability in the global wine industry. J. Wine Res..

[71] Hull C.E., Rothenberg S. (2008). Firm performance: The interactions of corporate social performance with innovation and industry differentiation. Strat. Manag. J..

[72] Broccardo L., Zicari A. (2020). Sustainability as a driver for value creation: A business model analysis of small and medium entreprises in the Italian wine sector. J. Clean. Prod..

[73] Schaltegger S., Lüdeke-Freund F., Hansen E.G. (2012). Business cases for sustainability: The role of business model innovation for corporate sustainability. Int. J. Innov. Sustain. Dev..

[74] Zhang N., Lin X., Yu Y., Yu Y. (2020). Do green behaviors improve corporate value? An empirical study in China. J. Clean. Prod..

[75] Dressler M., Paunovic I. (2021). A Typology of Winery SME Brand Strategies with Implications for Sustainability Communication and Co-Creation. Sustainability.

[76] Nilssen R., Bick G., Abratt R. (2018). Comparing the relative importance of sustainability as a consumer purchase criterion of food and clothing in the retail sector. J. Brand Manag..

[77] Castro V.A., Giraldi J.D.M.E. (2018). Shared brands and sustainable competitive advantage in the Brazilian wine sector. Int. J. Wine Bus. Res..

[78] Rinaldi S., Bonamente E., Scrucca F., Merico M.C., Asdrubali F., Cotana F. (2016). Water and Carbon Footprint of Wine: Methodology Review and Application to a Case Study. Sustainability.

[79] Iannone R., Miranda S., Riemma S., De Marco I. (2016). Improving environmental performances in wine production by a life cycle assessment analysis. J. Clean. Prod..

[80] Scrucca F., Bonamente E., Rinaldi S., Muthu S.S. (2018). Carbon Footprint in the Wine Industry. Environmental Carbon Footprints.

[81] Aivazidou E., Tsolakis N. (2020). A water footprint review of Italian wine: Drivers, barriers, and practices for sustainable stewardship. Water.

[82] Bonamente E., Scrucca F., Rinaldi S., Merico M.C., Asdrubali F., Lamastra L. (2016). Environmental impact of an Italian wine bottle: Carbon and water footprint assessment. Sci. Total. Environ..

[83] Dede D., Didaskalou E., Bersimis S., Georgakellos D. (2020). A Statistical Framework for Assessing Environmental Performance of Quality Wine Production. Sustainability.

[84] Zamagni A. (2012). Life cycle sustainability assessment. Int. J. Life Cycle Assess..

[85] Falcone G., De Luca A.I., Stillitano T., Strano A., Romeo G., Gulisano G. (2016). Assessment of Environmental and Economic Impacts of Vine-Growing Combining Life Cycle Assessment, Life Cycle Costing and Multicriterial Analysis. Sustainability.

[86] Arzoumanidis I., Raggi A., Petti L. (2014). Considerations When Applying Simplified LCA Approaches in the Wine Sector. Sustainability.

[87] Hermann B., Kroeze C., Jawjit W. (2007). Assessing environmental performance by combining life cycle assessment, multi-criteria analysis and environmental performance indicators. J. Clean. Prod..

[88] Rowland-Jones R., Pryde M., Cresser M. (2005). An evaluation of current environmental management systems as indicators of environmental performance. Manag. Environ. Qual. Int. J..

[89] Gierling F., Blanke M. (2021). Carbon reduction strategies for regionally produced and consumed wine: From farm to fork. J. Environ. Manag..

[90] Peters G.P., Hertwich E. (2008). CO_2_ Embodied in International Trade with Implications for Global Climate Policy. Environ. Sci. Technol..

[91] Arjen Y., Hoekstra T., Wiedmann O. (2014). Humanity’s unsustainable environmental footprint. Science.

[92] Ayuda M.-I., Esteban E., Martín-Retortillo M., Pinilla V. (2020). The Blue Water Footprint of the Spanish Wine Industry: 1935–2015. Water.

[93] Duarte R., Pinilla V., Serrano A. (2014). The Spanish Food Industry on Global Supply Chains and Its Impact on Water Resources. Water.

[94] Duarte R., Pinilla V., Serrano A. (2019). Long Term Drivers of Global Virtual Water Trade: A Trade Gravity Approach for 1965–2010. Ecol. Econ..

[95] Török Á., Jantyik L., Maró Z., Moir H. (2020). Understanding the Real-World Impact of Geographical Indications: A Critical Review of the Empirical Economic Literature. Sustainability.

[96] Lučić S. (2018). EU trademarks for wine which contains indications of geographical origin. Ekon. Poljopr..

[97] López-Bayón S., Fernández-Barcala M., González-Díaz M. (2020). In search of agri-food quality for wine: Is it enough to join a geographical indication?. Agribusiness.

[98] Deconinck C., Swinnen J. (2014). The political Economy of Geographical Indications.

[99] Meloni G., Swinnen J. (2018). Trade and Terroir: The Political Economy of the World’s First Geographical Indications.

[100] Belmin R., Casabianca F., Meynard J.M. (2018). Contribution of transition theory to the study of geographical indications. Environ. Innov. Soc. Transit..

[101] Allaire G., Sylvander B., Sylvander B., Barham E. (2011). Globalization and Geographical Indications. Geographical Indications and Globalization in Agrofood Supply Chains.

[102] Addor F., Grazioli A. (2005). Geographical Indications beyond Wines and Spirits. J. World Intellect. Prop..

[103] Belletti G., Sylvander B., Barjolle D., Arfini F. (1999). Origin labelled products, reputation and heterogeneity of firms. The Socio-Economics of Origin Labelled Products in Agro-Food Supply Chains: Spatial, Institutional and Co-Ordination Aspects.

[104] Dogana B., Gokovalib U. (2012). Geographical indications: The aspects of rural development and marketing through the traditional products. Procedia Soc. Behav. Sci..

[105] Sylvander B., Allaire G., Belletti G., Marescotti A., Barjolle D., Thévenod-Mottet E., Tregear A. (2006). Qualité, origine et globalisation: Justifications générales et contextes nationaux, le cas des indications géographiques. Rev. Can. Sci. Régionales.

[106] Thévenod-Mottet E., Lockie S., Carpenter D. (2010). Geographical indications and biodiversity. Agriculture, Biodiversity and Markets: Livelihoods and Agroecology in Comparative Perspective.

[107] Martini A., Frederichi F., Rosini G., Fattal B., Katzenelson E., Federici F. (1980). A new approach to the study of yeast ecology on natural substrates. Can. J. Microbiol..

[108] Cordero-Bueso G., Arroyo T., Serrano A., Tello J., Aporta I., Vélez M.D., Valero E. (2011). Influence of the farming system and vine variety on yeast communities associated with grape berries. Int. J. Food Microbiol..

[109] Longo E., Cansado J., Agrelo D., Villa T.G. (1991). Effect of climatic conditions on yeast diversity in grape musts from northwest Spain. Am. J. Enol. Vitic..

[110] Jackson D.I., Lombard P.B. (1993). Environmental and management practices affecting grape composition and wine quality-a review. Am. J. Enol. Vitic..

[111] Haeck C., Meloni G., Swinnen J. (2018). The Value of Terroir: A Historical Analysis of the Bordeaux and Champagne Geographical Indications.

[112] Costanigro M., Scozzafava G., Casini L. (2017). Vertical Differentiation, Perceptions Restructuring, and Wine Choices: The Case of the Gran Selezione in Chianti Wines.

[113] Cross R., Plantinga A.J., Stanvis R.N. (2017). Terroir in the new world: Hedonic estimation of vineyard sales price in California. J. Wine Econ..

[114] Bartlett G. (2020). An Empirical Analysis of the Effect of Sub-Divisions of American Viticultural Areas on Wine Prices: A Hedonic Study of Napa Valley. J. Wine Econ..

[115] Viju C., Yeung M.T., Kerr W.A. (2012). Geographical Indications, Barriers to Market Access and Preferential Trade Agreements.

[116] Galbreath J. (2017). Drivers of Green Innovations: Evidence from the Wine Industry.

[117] Yi-Ming W., Meng J., Guan D., Shan Y., Song M., Wei Y.-M., Liu Z., Hubacek K. (2017). Chinese CO_2_ emission flows have reversed since the global financial crisis. Nat. Commun..

[118] Wang Q., Hubacek K., Feng K., Guo L., Zhang K., Xue J., Liang Q.-M. (2019). Distributional impact of carbon pricing in Chinese provinces. Energy Econ..

[119] Meng B., Liu Y., Andrew R., Zhou M., Hubacek K., Xue J., Peters G., Gao Y. (2018). More than half of China’s CO_2_ emissions are from micro, small and medium-size enterprises. Appl. Energy.

[120] Timmer M.P., Dietzenbacher E., Los B., Stehrer R., De Vries G.J. (2015). An Illustrated User Guide to the World Input-Output Database: The Case of Global Automotive Production. Rev. Int. Econ..

[121] Ministerio de Agricultura, Pesca y Alimentación (MAPA) Datos de Las Denominaciones de Origen Protegidas de Vinos (DOPs). https://www.mapa.gob.es/es/alimentacion/temas/calidaddiferenciada/informedops2017-2018modif_tcm30-513739.pdf.

